# Bibliographical Notices

**Published:** 1846-12

**Authors:** 


### BIBLIOGRAPHICAL NOTICES.

*C. Hering's Domestic Physician. Third American edition, comprising the former edition of the Homœopathist, or Domestic Physician; revised, with additions from the Author's manuscript of the fifth German edition, together with the additions of Drs. Goullon, Gross, and Stapf; to which is added a Chapter on the Diseases of Women.* 12mo. pp. 412. Philadelphia, 1845.

We have often had occasion to refer to the absurdities of homœopathy, not only as a theoretical but practical subject. The remarks we have made have usually, however, been of a general character. Never have we noticed any production, at length, from a professed homœopathist; but we cannot resist the impulse to lay before them the views, principles (save the mark!) and treatment of the great apostle of Hahnemannism in this country.

Of this work—it is proper to declare in the outset—the British Journal of Homœopathy has spoken in terms of the most elevated praise, and an extract from it is placed by the publisher immediately after the preface. Of the practical *tact* of Dr. Hering the reviewer speaks, doubtless, in terms of merited encomium; every thing, he says, "is written down in plain language, and arranged with a *methodic simplicity indicative of a scientific mind.*" "What pleases us most," he adds, "about the book, is the *sound common sense* which pervades the whole of it;" and he concludes—"We cannot but regard this book as a very great acquisition; and should be most unwilling to include it in the sweeping censure we felt it our duty to pronounce on Domestic Treatises generally. Besides, Dr. Hering has indicated his claim to write a domestic treatise, by having *graduated in authorship*, by the publication of many admirable *strictly scientific* papers, and having established his reputation as a successful practitioner, well entitled to give advice. *His book derives its name from him; he does not seek a name from it.* We heartily recommend this little work as a most useful and innocent family counsellor; and should not be surprised if, in time, it became, both for this country and America, the Homœopathic Buchan." p. v.

Thus, the "Domestic Physician," of Dr. Hering, is endorsed by his compeers on the other side of the Atlantic, who cheerfully award him all praise as the *magnus Apollo*, in America, of homœopathy.

Let us then note, from his own pen, what are his claims to profound philosophy, to "graduation in authorship," to "plain language" and methodic simplicity of arrangement, "indicative of a scientific mind." With this view, we shall permit him, as far as practicable, to speak for himself, in order that we may not misrepresent him. His first chapter is on affections of the mind,—"sudden emotions" engaging his attention first. The very first remark indicates—to say the least of it—commendable prudence, and great care to avoid error. Sudden emotions, he tells us, " are often attended with injurious consequences, *which appear immediately or some time after,*"—inevitable results, we should presume, if they occur at all! For every emotion Dr. Hering has a remedy. Thus he says, " If anger and vexaticn produce mental alienation, give Platina. When little children get into so violent a rage as to lose their breath, or fall into convulsions, give Chamomile. If they shriek and weep violently, with frequent attacks of coughing, give Arnica. If they continue to cry, and will not be pacified, give Belladonna ; and if this does no good, give Hep. [Hepar sulphuris calcis?] ; the latter medicine but once." p. 7.

The following directions for rheumatic pains are rich, and eminently "scientific,"—to say nothing of the elegance and precision of the diction.

" When such pains result from cold, and the parts feel uneasy so as to require constant change of position, every thing feeling as if too hard, and the limbs as if palsied, *the patient complaining when a person is walking across the room or is approaching him,* give Arnica. But if the pains are attended with fever, give Aconitum napellus every three hours, until the fever abates, and then, after several hours, give Arnica. In many cases, it will be well to give Arnica and Aconitum alternately every six hours till the patient is relieved from his acute suffering.

If the rheumatic pains are worse when lying down, and at night, accompanied by lameness or coldness of the limbs, with pale swelling or burning in the feet, or with redness and swelling of the big toe, with a stiff neck, the skin very dry, or offensive perspiration, which affords no relief, give Dulc.; and if this no longer affords relief, Merc. viv.

When the same symptoms return after every cold, with uneasiness at the approach of other persons, or when attempting to swallow ; when it is worse when sitting or lying, but better when walking up and down, with swelling in the big toe, much rending, burning, and throbbing in the head, give Phosph. acid. If accompanied by swelling of the knees, lumps on the joints, and the hands and fingers, give Sulph. and, if this does not answer, Calc." p. 13.

Under headache, (p. 19.) we are told " when the sick person is very peevish in the morning, cannot bear his clothes, is more passionate and cross than plaintive and desponding, and apprehensive of future evil, give Bry." [Bryonia alba.]

The treatment of "swelling of the nose," is thus lucidly described:

"When the nose is swelled in consequence of a blow, [qu. of the nose?] or when it [qu. the nose?] happens without any apparent cause, accompanied [qu. the nose?] by itching, pain in the superior part of the bone, as if after a blow, give Arn. When attended by catarrh, when the orifice of the nostril is swelled and sore, with redness, heat, and pains, *sometimes running far in*, with burning, pricking and dryness; the smell sometimes very sensitive, sometimes too weak, give Bell.; and where it does not suffice, Hep. after it. When the catarrh is running, [qu. whither?] *very watery*, makes the nose sore, which is swelled, red, and shines, with itching, pains in the bone when pressed upon, [qu. the pains?] it is better to begin with Merc. viv., and to give Hep. afterwards, or perhaps Bell. Persons who have taken much calomel should first take Hep., and afterwards Merc. viv. Bry. sometimes is of service in tedious, painful swelling; or when black specks are on the nose, Sulph.; for red specks, Phosph. acid.; for warts, Caus.; when the point of the nose is red, Rhus; when the nose is of a coppery red, with craving for spirituous liquor, Ars." p. 185.

The following is a sage homœopathic principle :—

"Nothing should be given for constipation in childbed, *even though it last a fortnight—as it is at all times a very good sign, and promotes the strength of the patient.* After a fortnight one of the remedies recommended under constipation, particularly Bry., may be given; if it produces no effect in twelve hours, try it once more, and if, after the second dose, no evacuation takes place in a couple of hours, give an injection of luke warm water." p. 339.

This reminds us of a friend affected with constipation, who determined to trifle no longer, but "to take the bull by the horns"—to use his own expression—and therefore took a seidlitz powder!! But what shall we say of the success of homœopathic treatment in small pox?

"Small pox is so easily cured by one or a couple of doses of Sulph. or Rhus, that this disease should no longer excite any uneasiness [!!] In the beginning, when it does not appear on the skin, give Ipec. and Tart. alternately." p. 361.

Of erysipelas he says, "It is an old rule that nothing fat and damp must be applied in this complaint; *every salve and oint-*

*ment is dangerous, and no rational doctor of the old school will countenance it.*"

But the armamentarium medicum for toothache is the richest of all. The observations on this "Scotch hell of a' diseases," occupy upwards of sixteen pages, of which we shall extract only a portion.

"TOOTHACHE, from cold: Acon., Ign., Bell., Merc. viv., Puls., Nux vom., Cham., Dulc., Rhus, Hyos.; cold makes it worse, Merc. viv., Phosph. acid., Sulph., Ars., Ant., Calc.; aggravated by cold air, Bell., Merc. viv., Staph., Sulph., Hyos.; by cold water, Bry., Nux vom, Sulph., Ant., Calc.; by drinking cold drinks, Merc. viv., Puls., Staph., Nux vom., Cham., Sulph., Calc.; by cold washing, Merc. viv., Sulph., Calc.; drawing of cold air into the mouth, Bell., Merc. viv., Phosph. acid., Bry., Nux vom., Sulph.

Better when applying a cold hand to it, Rhus; cold water for a moment, Bry.; fingers dipped in water, Cham.

Worse in the open air, Bell., Staph., Nux vom., Chin., Sulph., Rhus; in the wind, Puls., Rhus; from draught, Chin., Sulph., Calc.; worse when in the room, Cham., Sulph., Ant. crud.

Better in the open air, Puls., Bry., Hep.

Worse from something hot, Bell., Phosph. acid.; from warmth, Cof., Puls., Bry., Cham., Sulph.; drinking warm, Merc. viv., Nux vom., Cham.; eating warm, Bry.; in a warm room, or where it is warm in general, Puls., Phosph. acid., Hep., (Cham., Sulph.;) when warm in bed, Bell., Merc. viv., Puls., Phosph. acid., Bry., Cham.

Better from heat, Merc. viv., Nux vom., Sulph., Ars., Rhus.

Worse from smoking, Ign., Bry., Chin.; better from the same, Merc. viv.

Worse from drinking coffee, Ign., Nux vom., Cham.; from drinking wine, Nux vom.; from drinking wine of any kind, Cham.

Worse when eating, Bell., Merc. viv., Puls., Phosph. acid., Staph., Bry., Hep., Sulph., Carb. veg.; after eating, Cof., Ign., Bell., Staph., Bry., Nux vom., Cham., Sulph., Ant. crud.; some time after eating, Bell.

Worse when moving the mouth, Nux vom. and Cham.; when chewing, Merc. viv., Staph., Bry., Nux vom., Sulph., Ars., Carb. veg., Hyos.; when biting, Cof., Bell., Puls., Nux vom., Hep., Sulph., Rhus; when striking the teeth together, Hep., Chin.; better from it, Cof., Chin., Ars.

Worse when touched, Bell., Merc. viv., Phosph. acid., Staph., Bry., Nux vom., Arn., Hep., Ars., Carb. veg.; when touched by the tongue, Ign., Merc. viv., Chin., Carb. veg.; better on being pressed, Bell., Puls., Chin., Rhus; better when rubbed, Merc.

viv.; worse when picking, Puls.; better when they bleed, Bell.; worse when in motion, Bry., Nux vom., Chin.; better when at rest, Bry.; worse when sitting, Puls., Rhus; better when sitting up in bed, Merc. viv., Ars., Rhus; worse when lying down, Ign.; on the side where it pains, Ars.; on the other side, Bry.; better when lying down, Merc. viv.; better when lying on the side on which is the pain, Bry.; when worse at night, Cof., Bell., Merc. viv., Puls., Phosph. acid., Staph., Bry., Cham., Hep., Sulph., Ars., Sil., Rhus, Calc.; in the evening, in bed, Merc. viv., Ant. crud.; worse when going to sleep, Ars.; better, Merc. viv., Staph.; worse when awaking, Bell., Nux vom., Carb. veg.; in the morning, Ign., Merc. viv., Puls., Phosph. acid., Staph., Bry., Nux vom., Chin., Sulph., Ars., Hyos.; in the forenoon, Puls., Sulph., Carb. veg.; worse in the afternoon, Merc. viv., Puls., Nux vom., Sulph.; toward evening, Puls.; in the evening, Ign., Bell., Merc. viv., Puls., Bry., Nux vom., Sulph., Ant. crud., Rhus.

Worse when there is a noise, Calc.; when addressed by others, Bry., Ars.; with vexation, Rhus; when thinking, Bell., Nux vom.; when reading, Ign., Nux vom." p. 245.

We scarcely know what to admire most in these extracts, which are fair samples of the contents of the book, and indeed of all homœopathic books,—the immense extent of observation which must have been necessary for the adaptation of the drugs to the special morbid conditions, or the confident tone with which such fancied adaptation is promulged. Where so many agents are used, and time is permitted to elapse in their administration, it would be strange, indeed, if improvement did not take place, but it would be still more strange, if the improvements were induced by the prescribed agent.

Our readers have thus had a full opportunity for judging of homœopathy, from one of its *celebrated* supporters. Can they fail to consider it eminently meriting all that we have hitherto said of it. Never has there been a tissue of more silly conceits, since the creation of the world. Perhaps it is as harmless as any that have been devised, and as the people must be deceived, and a tub must be constantly thrown out to amuse the whale, it may perhaps be tolerated with as much equanimity as any. Its decadency has already, however, commenced, and before long we shall hear of it as a delusion that once existed, but we have little hopes that succeeding delusions will be less exceptionable.

*Lectures on Natural and Difficult Parturition.* By Edward William Murphy, A. M., M. D. Professor of Midwifery, University College, London; Obstetric Physician, University College Hospital, etc. 8vo. pp. 281. Samuel S. & William Wood, New York. 1846.

These lectures, the author informs us, "are the substance of those that have been given on the subject of Natural and Difficult Parturition in University College," London; and are published "in order that his pupils, recalling to their minds the lessons they have received, may be enabled to examine the principles and precepts delivered to them with more care and attention than could be done from the evanescent impressions of an oral lecture." They do not appear to be published exactly in the order delivered by the Professor, and, although the style of address adapted to such occasions is preserved, in other respects we can discover little of the form of lectures. The long, unbroken, and elaborately constructed sentences—without episode, amplification, or varied illustration—which occupy its pages, however elegant in an essay, would render the discourses of a public lecturer excessively dry and ludicrously pedantic—qualities which we are sure cannot characterize the lectures of the distinguished author, and we do not see why he has preserved the title when the form has been so manifestly departed from. However, "the substance" is the same, and that is the important part.

The book is divided into *thirteen* "Lectures." The 1st is on the "*bones of the pelvis and pelvis collectively—measurements of the pelvis and fœtal head;* 2nd. *Modes of measuring the pelvis;* 3rd and 4th. *Mechanism of parturition;* 5th and 6th. *Management of natural labour;* 7th. *Difficult labours;* 8th, 9th and 10th. *Laborious labours*; 11th. *Obstetric operations*—the forceps; 12th. *Obstetric operations;* 13th. *Obstetric instruments.*"

On the subject of the pelvis, its measurements, etc., we discover nothing particular to remark, except the importance given by the author to the measurements of the *cavity* of the pelvis as well as its *straits*, which, he thinks, and we believe justly, is too much overlooked in obstetrical treatises.

A distinction is likewise made between *deviations* from the ordinary measurements, such as greater or lesser size, etc., from unusual or too early arrest of development, and *deformities* proceeding from disease; which, so far as parturition is concerned, appears to us "a distinction without a difference."

Of the deviations from disease, he recognizes three varieties, to wit, 1st, the *Ovate;* the oval form of the brim arising from the closer approximation of the promontory of the sacrum to the symphysis pubis. The 2nd, or *cordiform;* "that is, it resembles the ace of hearts; but when the distortion is great, it approaches much nearer the letter Y. The 3rd, "*Obliquely ovate pelvis of Naegele.*" One side of the pelvis of its usual shape, and the opposite running in almost a straight line.

"These three are the leading varieties we meet with in the *deformed* pelvis; but from the two former there are numerous deviations, still, however, preserving their specific characters. The first is generally described as the deformity from *rickets*, and as being caused in infancy; the second, as deformity from mollities ossium, and produced in the adult by that peculiar disease. The third variety has been known only within the last few years; and we are indebted for our knowledge of it principally to the distinguished Naegele." For the production of this variety of distortion he adopts the theory of Dr. Knox, which attributes it to arrest of developement on *one side* of the pelvis before ossification is complete, the other side progressing to its proper size; which is in all probability the true explanation.

The directions of Prof. Murphy for the management of parturient cases, are generally in accordance with those of other distinguished teachers, and of course correct; but on some points we cannot quite agree with him. Thus, when the head of the child is arrested with *the face towards the pubis*, he remarks, "Sometimes the correction may be made with the finger, but it is preferable to introduce the vectis on the pubic side of the pelvis, and to rotate it gradually towards the sacral side." Now, when "*the face is towards the pubis,*" "to introduce the *vectis on the pubic side of the pelvis*" to rotate the head, would be to operate by pressing the instrument against the face, to the great danger of the child's features—perhaps to crush the nose or put out its eyes. In such cases we should prefer to introduce the hand into the vagina, and in the absence of a pain, push the head above the brim and change the presentation; or, if that were not practicable, to pass the vectis up *the side* of the pelvis, and apply it on the side of the head, a little in front of the ear, instead of placing it over the face, for the purpose of rotating the head.

Speaking of too great size and ossification of the child's head as a cause of difficult labour, the author observes: "As far as I have had the opportunity of judging, this kind of head is very often met with when the pelvis is in a similar condition—too much ossified."

No reason is assigned why this should be the case, nor can we conceive of any, and it is probable that in the cases referred to by the author, coincidence rather than cause was concerned. Where the pelvis is of full size, a large and well ossified head commonly passes without extraordinary difficulty, and the practitioner never seriously thinks of the peculiarity, and still less of there being any thing unusual about the pelvis.

On the rules to be observed in the employment of obstetrical instruments, and especially of the forceps, in difficult labours, Professor Murphy accords with the generality of British practitioners—or we might say, with Denman and Collins, rather than Hamilton and Burns; and not with Continental and American practitioners. The work is enriched with plates representing distorted pelves, some of the presentations, and various forms of instruments for performing obstetric operations, and concludes with a sort of *resumé* of all the practical precepts in the form of Aphorisms, after the manner of Denman. In fact, many of them are a reprint of those of that author, and none differ essentially from them.

Notwithstanding the few objections we have mentioned, the book is a decidedly good one—inculcating in the main sound doctrines, clearly and modestly expressed. Its saying nothing in relation to the diseases of females, either of the pregnant or non-pregnant state, will be an objection to those who desire a treatise on all those subjects within the compass of a single volume; but with others, and especially teachers who prefer a text book for their pupils which shall not embrace all they may have to communicate, this may be a recommendation.

The copy before us is a reprint of the London edition, and is creditable for its mechanical execution.

*Adulterations of various substances used in Medicine and the Arts, with the means of detecting them; intended as a Manual for the Physician, the Apothecary, and the Artisan.* By LEWIS C. BECK, M. D., Professor of Chemistry in Rutger's College, New Jersey, and in the Albany Medical College, etc. etc. 12mo. pp. 333. Samuel F. and William Wood. New York, 1846.

The design of this work is to exhibit the adulterations of various substances, being principally such as are used in Medicine and the Arts, and to describe the processes by which they may be detected.

The processes for the detection of these adulterations are usually quite simple, and may be easily followed even by the tyro in Chemistry; in a few cases, however, they are more difficult, and some knowledge of the details of the science, and some experience in the business of testing will be necessary. Throughout the work the author has endeavoured to render the descriptions of the processes recommended as plain and free from technicalities as possible.

Some of the substances treated of can hardly be classed with such as are "used in Medicine or the Arts," although it may be satisfactory to most persons to know how to detect their adulterations; such as, for instance, *Cider, Coffee, Chocolate, Mushrooms, Wheat-flour, Milk, &c.*

Besides a copious and well arranged *index,*—without which even a good book is scarcely worth owning,—the volume contains an appendix, treating of "operations and instruments employed in qualitative analysis," "preparation of tests or reagents," and "tables exhibiting the behaviour of some reagents with the more important metals, metallic oxides, and acids."

By the physician and the artisan, this little volume will be found to be highly convenient and useful as a manual on many subjects which concern them; whilst the miscellaneous character of its contents, its plain style, and freedom from technicalities, will render it acceptable to intelligent and scientific readers of every class.

*Minor Surgery ; or hints on the every day duties of the Surgeon.* By HENRY H. SMITH, M. D., Lecturer on the Practice of Surgery ; Fellow of the College of Physicians ; Member of the Philadelphia Medical Society, etc. Second Edition, with numerous additions. Illustrated by 227 engravings. 12 mo. pp. 384. E. Barrington, and G. D. Haswell. Philadelphia, 1846.

Works like this are not calculated for great display, and yet they are among the most useful of the many publications that come from the press. The young practitioner will find occasion for information on the subjects which occupy them a hundred times in the discharge of his professional duties, for once that he will be called on to perform the greater operations described in the more elaborate treatises on surgery. The neat application of bandages, proper construction and application of splints, and many other such matters, which are described and shown in the figures in this publication, are neither so simple nor so unimportant as the inexperienced are apt to imagine; and hence we look upon any and every means, calculated to give greater facility and tact in the performance of these duties, as particularly deserving of the young surgeon's attention.

The present work, it appears to us, is well adapted to give the requisite information on the subjects of which it treats. It is, of course, as all such must necessarily be, a compilation. A list of authors is given, whose works have been "consulted in connection with the present subject." It would have been better perhaps to have indicated, in their proper places, the authors from whom the particular contrivances and suggestions are derived, instead of the general acknowledgement which is given. The plan pursued is calculated to save trouble to the author, but it is at the expense of those to whose labors he is indebted for the materials of which his work is constructed, which, we are quite sure, Dr. Smith would not desire:—he is not without precedents enough, however, for the course he has adopted.

